# Comparison of fully-covered vs partially covered self-expanding metallic stents for palliative treatment of inoperable esophageal malignancy: a systematic review and meta-analysis

**DOI:** 10.1186/s12885-020-6564-6

**Published:** 2020-01-30

**Authors:** Chunmei Wang, Hua Wei, Yuxia Li

**Affiliations:** 1 Department of Thoracic and Cardiovascular Surgery, Huaihe Hospital of Henan University, Kaifeng, Henan 475000 People’s Republic of China; 2 Department of Laboratory, Huaihe Hospital of Henan University, 8 Baobei Road, Kaifeng, Henan 475000 People’s Republic of China

**Keywords:** Esophageal cancer, Meta-analysis, Palliative treatment, Dysphagia

## Abstract

**Background:**

This study aimed to compare clinical outcomes following placement of fully covered self-expanding metallic stents (FCSEMS) vs partially covered self-expanding metallic stents (PCSEMS) for palliative treatment of inoperable esophageal cancer.

**Methods:**

We searched PubMed, ScienceDirect, Embase, and CENTRAL (Cochrane Central Register of Controlled Trials) databases from inception up to 10th July 2019. Studies comparing clinical outcomes with FCSEMS vs PCSEMS in patients with inoperable esophageal cancer requiring palliative treatment for dysphagia were included.

**Results:**

Five studies were included in the review. Two hundred twenty-nine patients received FCSEMS while 313 patients received PCSEMS in the five studies. There was no difference in the rates of stent migration between FCSEMS and PCSEMS (Odds ratio [OR] 0.63, 95%CI 0.37–1.08, *P* = 0.09; I^2^ = 0%). Meta-analysis indicated no significant difference in technical success between the two groups (OR 1.32, 95%CI 0.30–5.03, *P* = 0.78; I^2^ = 12%). Improvement in dysphagia was reported with both FCSEMS and PCSEMS in the included studies. There was no difference between the two stents for obstruction due to tissue growth (OR 0.81, 95%CI 0.47–1.39, *P* = 0.44; I^2^ = 2%) or by food (OR 0.41, 95%CI 0.10–1.62, *P* = 0.20; I^2^ = 29%). Incidence of bleeding (OR 0.57, 95%CI 0.21–1.58, *P* = 0.28; I^2^ = 0%) and chest pain (OR 1.06, 95%CI 0.44–2.57, *P* = 0.89; I^2^ = 0%) was similar in the two groups. Sensitivity analysis and subgroup analysis of RCTs and non-RCTs produced similar results. The overall quality of studies was not high.

**Conclusion:**

Our results indicate that there is no difference in stent migration, and stent obstruction, with FCSEMS or PCSEMS when used for palliative treatment of esophageal malignancy.

## Background

With a 5-year survival rate of less than 20%, esophageal cancers are among the leading causes of cancer-related death worldwide [1]. At least 50% of all esophageal malignancies have incurable disease at presentation [2]. Palliative treatment aimed at reduction of dysphagia and improving oral intake; is the primary goal for such patients. ﻿Over the past few decades, endoscopically placed self-expandable metallic stents (SEMS) have become a treatment of choice for palliative management [3]. SEMS consist of a cylindrical metallic frame that exerts a self-expansive force until it reaches its maximum fixed diameter [4]. It thereby expands the narrowed esophageal passage, rapidly restoring luminal patency, maintaining nutritional intake and improving quality of life [5].

Although rarely used now, the uncovered SEMS introduced in the 1990s were associated with a high reintervention rate due to stent obstruction secondary to a tumor or inflammatory tissue in-growth [6]. To overcome this complication, fully-covered SEMS (FCSEMS) were introduced with an outer synthetic coating of silicone or polyurethane derivatives. The covering prevents embedding of the stent in the esophageal wall and tissue in-growth in the lumen [7]. The covering also stops the extravasation of ingested oral contents in cases of esophageal fistula. Another advantage is that FCSEMS can be easily removed under endoscopic and/or fluoroscopic guidance. However, the lack of embedding in the esophageal wall makes them prone to migration as compared to uncovered stents [4]. To derive benefits of both uncovered SEMS and FCSEMS, partially covered SEMS (PCSEMS) were designed [8]. The covering in the case of PCSEMS is limited only to the stent body while the proximal and distal flanges remain uncovered thereby promoting embedding in the esophageal wall. This feature is believed to reduce the incidence of stent migration [3].

To date, several clinical reports have been published demonstrating good clinical results with both FCSEMS [9, 10] and PCSEMS [8, 11]. However, literature comparing the clinical outcomes of the two stents is limited. Therefore, the present study was designed to systematically search the literature and analyze evidence comparing the clinical outcomes following placement of FCSEMS and PCSEMS for palliative treatment of inoperable esophageal cancer.

## Methods

### Search strategy

This systematic review and meta-analysis was conducted following the guidelines of the PRISMA statement (Preferred Reporting Items for Systematic Reviews and Meta-analyses) [12] and Cochrane Handbook for Systematic Reviews of Intervention [13]. We searched PubMed, ScienceDirect, Embase, and CENTRAL (Cochrane Central Register of Controlled Trials) databases from inception up to 10th July 2019. Search items used were: “esophageal cancer”; “esophageal dysphagia”; “esophagus”; “malignancy”; “stent”; “metallic stent” and “palliative treatment”. The search strategy for PubMed and ScienceDirect database is presented in Table 1. Additionally, references of included studies and review articles on the subject were analyzed for the identification of any additional studies. Two reviewers independently performed the literature search. Citations were initially screened at the title and abstract level. Full texts of selected articles were then analyzed for inclusion in the review. Disagreements were resolved by discussion.

**Table 1:** Search queries and results for PubMed and ScienceDirect database

Search	Query	Records found	
		PubMed	ScienceDirect
1	Search ((esophagus) AND malignancy) AND stent	753	69
2	Search (esophageal dysphagia) AND metallic stent	251	40
3	Search (esophageal dysphagia) AND palliative treatment	1293	104
4	Search (esophageal dysphagia) AND stent	1158	254
5	Search (esophageal cancer) AND metallic stent	398	51
6	Search (esophageal cancer) AND palliative treatment	3010	195
7	Search (esophageal cancer) AND stent	1821	312

### Inclusion criteria

We used the PICOS (Population, Intervention, Comparison, Outcome, and Study design) outline for including studies. We included randomized controlled trials (RCTs), quasi-RCTs, prospective/retrospective cohort studies conducted on adult patients with inoperable esophageal cancer requiring palliative treatment for dysphagia (*Population*); evaluating any kind of FCSEMS (*Intervention*); comparing it with any kind of PCSEMS (*Comparison*) and assessing any of the following variables: dysphagia scores, stent migration, stent obstruction or complications (*Outcomes*). We excluded studies conducted on benign esophageal lesions, studies utilizing irradiated stents and those with anti-reflux mechanisms, studies comparing uncovered stents with FCSEMS or PCSEMS. Additionally, we excluded non-English language studies, studies comparing less than 5 patients, duplicate reports, case series, and case reports.

### Data extraction and outcomes

Data were extracted from the included trials by two independent reviewers using an abstraction form. The following details were sourced: Authors, publication year, sample size, baseline and demographic details, type of SEMS used, dysphagia scores, technical success rates, stent migration, stent obstruction, and other complications. The authors were contacted via email for missing data.

The primary outcome was the incidence of stent migration. Secondary outcomes were technical success, improvement of dysphagia, the incidence of stent obstruction by tissue growth or food and other complications. Technical success was defined as the endoscopic placement of SEMS in the intended position. In all included studies, the recurrence of dysphagia was due to either stent migration or stent obstruction caused by tissue growth or food. To provide clarity on differences between the two stents, we did not pool data under the common heading of “recurrent dysphagia” but these variables were pooled separately under different causes of recurrence (stent migration, obstruction by tissue and obstruction by food). When multiple stents were compared in a study, data for all types of FCSEMS and PCSEMS were extracted.

### Risk of bias

For quality assessment of randomized controlled studies (RCTs), the Cochrane Collaboration risk assessment tool for RCTs was used [14]. Studies were rated as low risk, high risk, or unclear risk of bias for: random sequence generation, allocation concealment, blinding of participants and personnel, blinding of outcome assessment, incomplete outcome data, selective reporting, and other biases. The remaining studies were analyzed by the risk of bias assessment tool for non-randomized studies (RoBANS) [15]. Studies were rated as low risk, high risk, or unclear risk of bias for: Selection of participants, confounding variables, intervention measurements, blinding of outcome assessment, incomplete outcome data, selective outcome reporting.

### Statistical analysis

Review Manager (RevMan, version 5.3; Nordic Cochrane Centre [Cochrane Collaboration], Copenhagen, Denmark; 2014) was used for the meta-analysis. Outcomes were summarized using the Mantel-Haenszel Odds Ratios (OR) with a 95% confidence interval (CI). A random-effects model was used to calculate the pooled effect size. Heterogeneity was calculated using the I^2^ statistic. I^2^ values of 25–50% represented low, values of 50–75% medium and > 75% represented substantial heterogeneity. Sub-group analysis was conducted for RCTs and non-RCTs. A sensitivity analysis was performed to assess the contribution of each study to the pooled effect size by sequentially excluding individual studies one at a time and reinterpreting the pooled OR estimates for the remaining studies. Publication bias was not assessed due to limited studies included in the review.

## Results

**Fig. 1:**
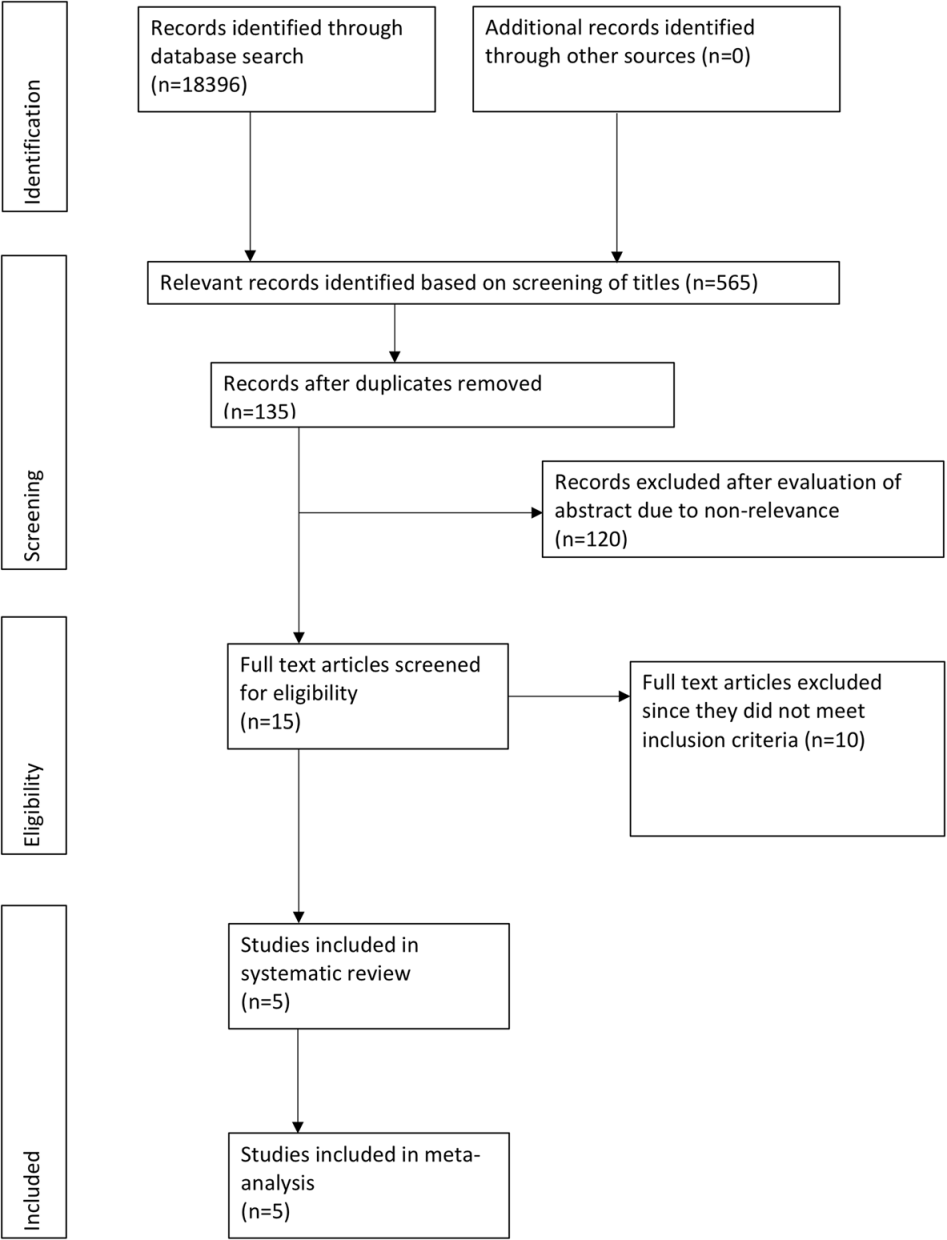
Flow chart of the study

A total of 18,396 records were identified by database searching (Fig. 1). Five hundred sixty-five relevant records were identified based on the screening of titles. After removing duplicates and non-relevant studies, fifteen articles were analyzed by their full-texts. Ten studies were excluded [16–25]. Detailed reasons for exclusion are presented in Table 2. Five articles [26–30] met the inclusion criteria and were analyzed in this systematic review and meta-analysis.

**Table 2:** Details of excluded studies

Study	Reason for exclusion
Uesato et al. [16]	Less than five patients in FCSEMS group
Battersby et al. [17]	Separate data not available for different stents used
Eickhoff et al. [18]	German language article
Gangloff et al. [19]	Used stents for benign growths
Sabharwal et al. [20]	Used stent with anti-reflux mechanism
Seven et al. [21]	Used stents for benign growths
Siersema et al. [22]	Overlapping data with Homs et al. [26]
Van Heel et al. [23]	Compared two PCSEMS
Wang et al. [24]	Compared uncovered vs covered stents
Wang et al. [27]	Compared irradiated stents

*FCSEMS* Fully-Covered Self Expanding Metal Stents, *PCSEMS* Partially-Covered Self Expanding Metal Stents

The characteristics of included studies are presented in Table 3. The mean age of patients in the included studies varied from 63.6 to 72.2 years. Three studies were RCTs [27–29], one was a retrospective review [30] while one was a prospective study [26]. A total of 229 patients received FCSEMS while 313 patients received PCSEMS across the five studies. The types of FCSEMS varied across trials. Two studies [28, 29] used the WallFlex fully-covered stent (Boston Scientific, Natick, Massachusetts, USA), while SX- ELLA® (ELLA-CS, Hradec Králové, Czech Republic), Niti-S stent (Taewoong Medical, Seoul, Korea) and Z-stent (Wilson-Cook Europe, Bjaeverskov, Denmark) were used in one study each. The use of Ultraflex® NG, (Boston Scientific, Natick, Massachusetts, USA) as PCSEMS was common with four studies [26–28, 30] reporting its use. In one study [26], two types of PCSEMS [Ultraflex® NG and Flamingo Wallstent (Microvasive/Boston Scientific)] were compared with the fully-covered Z-stent. We combined the data for both these PCSEMS for the meta-analysis. Dysphagia was scored in all studies according to the internationally used scoring system: score 0, able to consume a normal diet; score 1, dysphagia with certain solid foods; score 2, able to swallow semisolid soft foods; score 3, able to swallow liquids only; score 4, complete dysphagia. The malignancy was frequently located in the distal esophagus and cardia across all five studies.

**Table 3:** Characteristics of included studies

Author/year	Study type	Type of FCSEMS	Type of PCSEMS	Number of patients		Pre/post -Chemoradiotherapy		Dysphagia grade		Tumor location	
				FCSEMS	PCSEMS	PCSEMS	PCSEMS	FCSEMS	PCSEMS	FCSEMS	PCSEMS
Lárraga et al. [30]/2018	Retrospective	SX- ELLA® (ELLA-CS, Hradec Králové, Czech Republic)	Ultraflex® NG, (Boston Scientific, Natick, Massachusetts, USA)	21	29	Pre: 12 (57.14%) Post: 13 (61.9%)	Pre: 12 (41.37%) Post: 13 (44.82%)	Grade 3: 10 (47.61%) Grade 4: 11 (52.38%)	Grade 3: 13 (44.82%) Grade 4: 16 (55.17%)	P: 1 (4.76%) M: 5 (23.8%) D&C: 15 (71.44%)	P: 1 (3.45%) M: 9 (31%) D&C: 19 (65.55%)
Didden et al. [29]/2018	RCT	WallFlex fully covered stent (Boston Scientific, Natick, Massachusetts, USA)	WallFlex partially covered stent (Boston Scientific, Natick, Massachusetts, USA)	48	49	Pre: 41 (85.41%) Post: 5 (10.41%)	Pre: 41 (85.41%) Post: 5 (10.41%)	2.8 ± 0.7	2.8 ± 0.8	P: 8 (16.67%) M: 13 (27.08%) D&C: 27 (56.25%)	P: 1 (20.4%) M: 8 (16.33%) D&C: 27 (55.1%)
Persson et al. [28]/2017	RCT	WallFlex fully covered stent (Boston Scientific, Natick, Massachusetts, USA)	Ultraflex® NG, (Boston Scientific, Natick, Massachusetts, USA)	48	47	NR	NR	2 ± NR	3 ± NR	NR	NR
Verschuur et al. [27]/2008	RCT	Niti-S stent (Taewoong Medical, Seoul, Korea)	Ultraflex® NG, (Boston Scientific, Natick, Massachusetts, USA)	42	42	Pre:11 (26.19%) Post: 15 (35.71%)	Pre:11 (26.19%) Post: 15 (35.71%)	3 ± NR	3 ± NR	M: 12 (28.57%) D&C: 30 (71.43%)	M: 10 (23.8%) D&C: 32 (71.43%)
Homs et al. [26]/2004	Prospective	Z-stent (Wilson-Cook Europe, Bjaeverskov, Denmark)	Ultraflex® NG, (Boston Scientific, Natick, Massachusetts, USA) And Flamingo Wallstent (Microvasive/Bos- ton Scientific)	70	Ultraflex:75 Flamingo:71	Pre: 14 (20%) Post: 5 (7.14%)	Ultraflex: Pre: 17 (22.66%) Post: 6 (8%) Flamingo: Pre: 23 (32.4%) Post: 4 (5.63%)	3.2 ± 0.5	Ultraflex: 3 ± 0.7 Flamingo: 3.2 ± 0.5	M: 12 (17.14%) D&C: 58 (82.85%)	Ultraflex: M: 15 (20%) D&C: 60 (80%) Flamingo: M: 14 (19.72%) D&C: 57 (80.28%)

*FCSEMS* Fully covered- Self expanding metallic stents, *PCSEMS* Partially covered- Self expanding metallic stents, *RCT* Randomized controlled study, *P* proximal esophagus, *M* mid-esophagus, *D&C* Distal esophagus and cardia, *NR* Not reported

Data reported as Mean ± Standard Deviation or Number (percentage)

### Outcomes

**Fig. 2:**
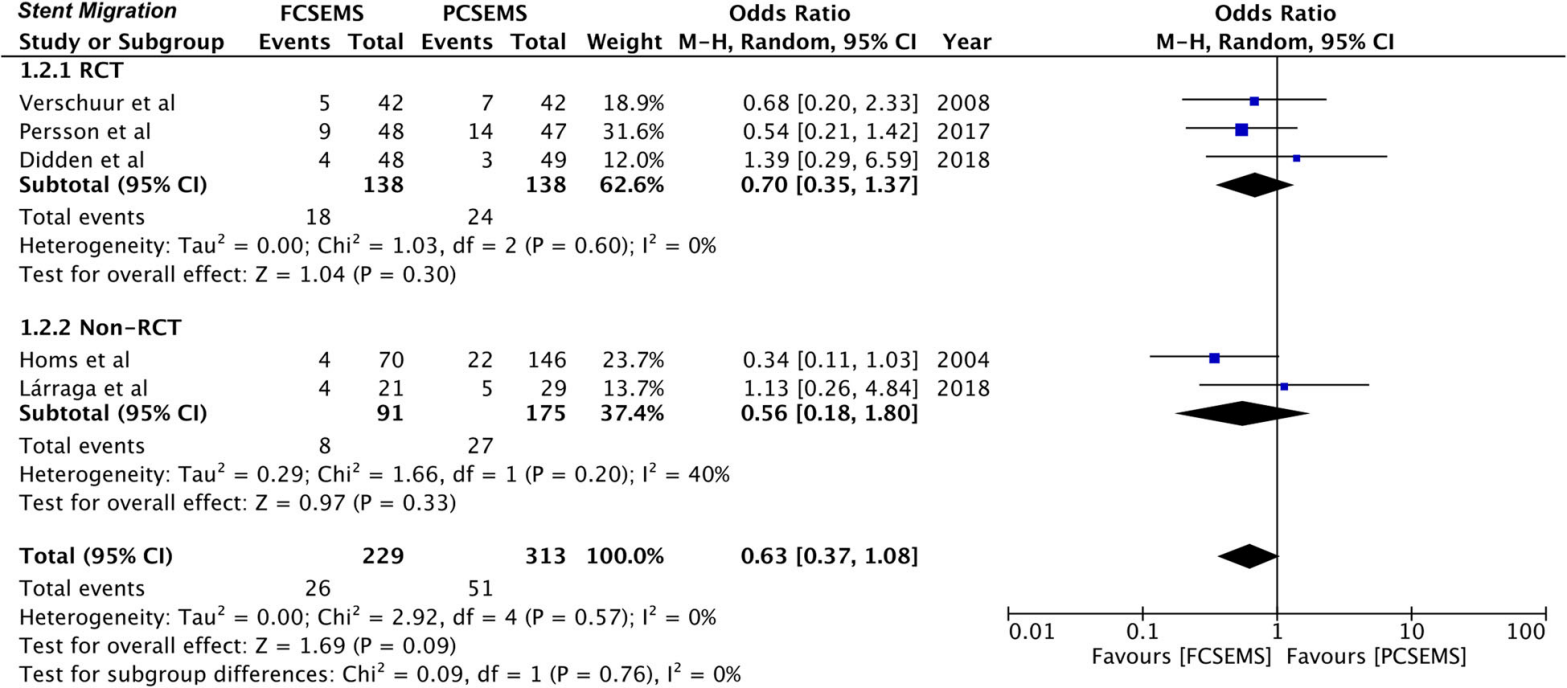
Forrest plot for stent migration

Outcomes of included studies are presented in Table 4. Data on stent migration was reported by all five studies [26–30]. Meta-analysis indicated no statistically significant difference in the rates of stent migration between FCSEMS and PCSEMS (OR 0.63, 95%CI 0.37–1.08, *P* = 0.09; I^2^ = 0%) (Fig. 2). Results were similar for sub-group analysis of RCTs (OR 0.70, 95%CI 0.35–1.37, *P* = 0.30; I^2^ = 0%) and non-RCTs (OR 0.56, 95%CI 0.18–1.80, *P* = 0.33; I^2^ = 40%) (Fig. 2).

**Table 4:** Outcomes of included studies

Outcome	Lárraga et al. [30]		Didden et al. [29]		Persson et al. [28]		Verschuur et al. [27]		Homs et al. [26]		
	FCSEMS *N* = 21	PCSEMS *N* = 29	FCSEMS *N* = 48	PCSEMS *N* = 49	FCSEMS *N* = 48	PCSEMS *N* = 47	FCSEMS *N* = 42	PCSEMS *N* = 42	FCSEMS *N* = 70	PCSEMS (Ultraflex) *N* = 75	PCSEMS (Flamingo) *N* = 71
Technical success	21 (100%)	29 (100%)	48 (100%)	47 (95.91%)	45 (93.75%)	43 (91.48%)	40 (95.23%)	42 (100%)	NR	NR	NR
Stent Migration	4 (19.04%)	5 (17.24%)	4 (8.33%)	3 (6.12%)	9 (18.75%)	14 (29.78%)	5 (11.9%)	7 (16.66%)	4 (5.71%)	17 (22.66%)	5 (7.04%)
Stent obstruction by tumor	0	5 (17.24%)	5 (10.41%)	7 (14.28%)	0	2 (4.25%)	10 (23.81%)	13 (30.95%)	11 (15.71%)	7 (9.33%)	12 (16.9%)
Stent obstruction by food	2 (9.52%)	2 (6.9%)	0	1 (2%)	0	5 (10.63%)	1 (2.38%)	0	1 (1.42%)	10 (13.33%)	5 (7.04%)
Chest Pain	1 (4.76%)	2 (6.9%)	9 (18.75%)	9 (18.36%)	NR	NR	2 (4.76%)	1 (2.38%)	NR	NR	NR
Bleeding	0	1 (3.45%)	4 (8.33%)	5 (10.2%)	NR	NR	2 (4.76%)	5 (11.9%)	NR	NR	NR

*FCSEMS* Fully covered- Self expanding metallic stents, *PCSEMS* Partially covered- Self expanding metallic stents, *NR* Not reported

**Fig. 3:**
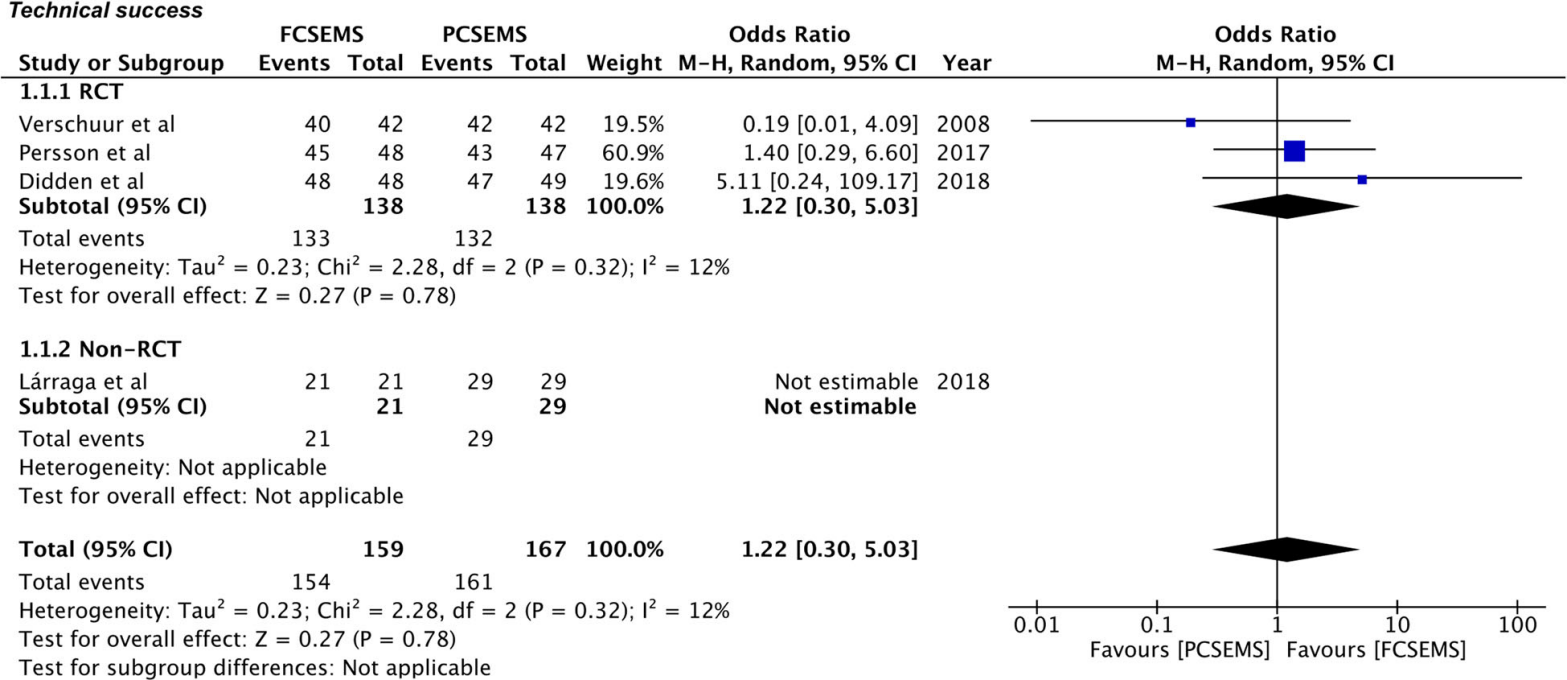
Forrest plot for technical success

Four studies [27–30] reported data on technical success. Pooled data of 159 patients in the FCSEMS group and 167 patients in the PCSEMS group indicated no significant difference between the two groups (OR 1.22, 95%CI 0.30–5.03, *P* = 0.78; I^2^ = 12%) (Fig. 3). Since the only non-RCT [30] included in this analysis reported 100% success with both FCSEMS and PCSEMS, the pooled estimate is effectively an analysis of RCTs only.

Definitions of improvement of dysphagia varied across studies. Hence, data were not pooled for a meta-analysis and are presented in a descriptive form. Lárraga et al. [30] defined improvement of dysphagia as reduction of dysphagia score of equal to or greater than 2 grades. Improvement was reported in 90.2%of patients with FCSEMS and 89.6% of patients with FCSEMS with no statistical significant difference between the two groups. Didden et al. [29] reported improvement of dysphagia as at least 1 point reduction in dysphagia score. With 83% success with FCSEMS and 88% success with PCSEMS, there was no difference between the two stents. Persson et al. [28] compared pre and post dysphagia scores using three instruments; the Watson dysphagia score [31], the Ogilvie score [32] and a symptom-oriented quality of life instrument that has a module that captures information regarding swallowing difficulties (QLQ-OG25) [33]. No statistical significant difference was seen between the two groups with any scoring instrument. Verschuur et al. [27] reported an improvement of dysphagia scores from a median of 3 (liquids only) to 1 (ability to eat some solid food) with both FCSEMS and PCSEMS.

**Fig. 4:**
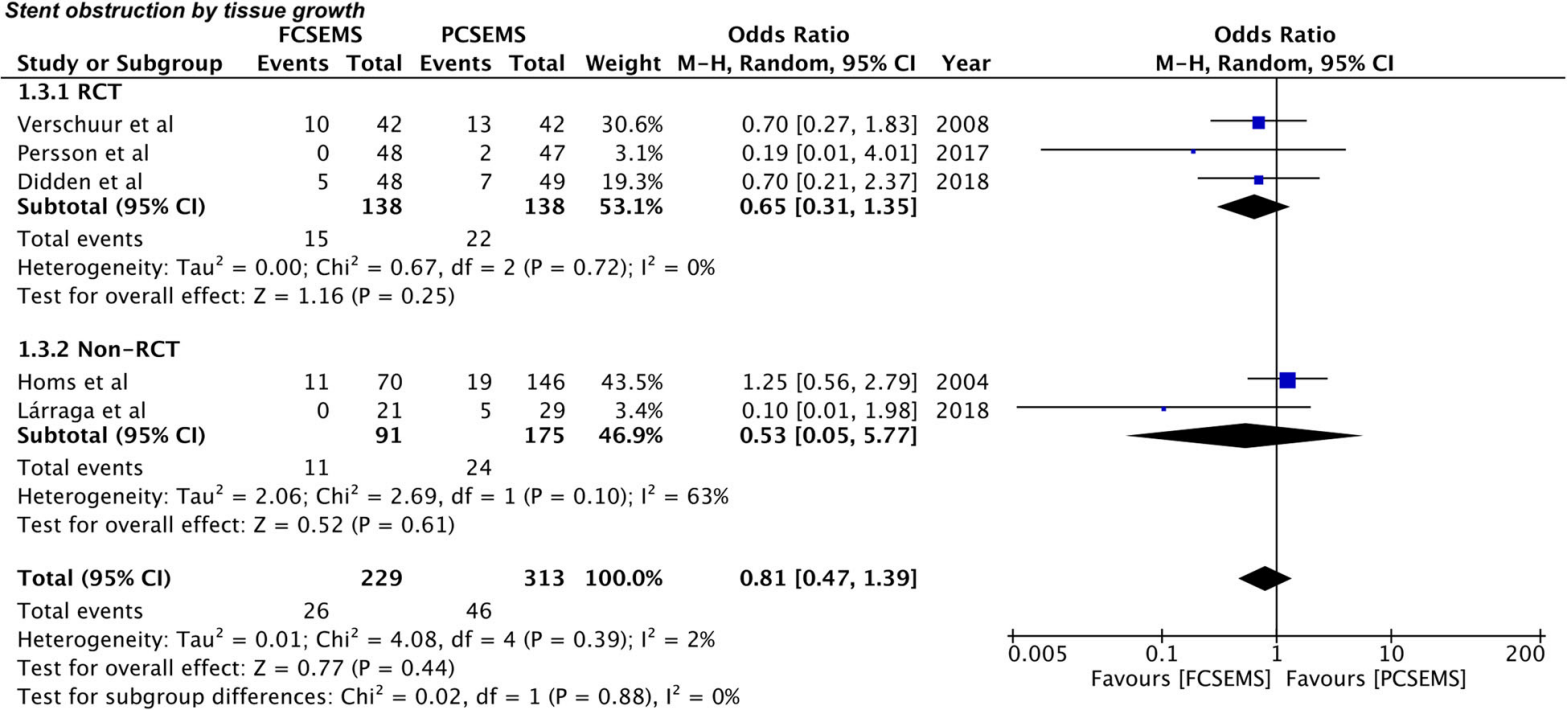
Forrest plot for stent obstruction by tissue growth

**Fig. 5:**
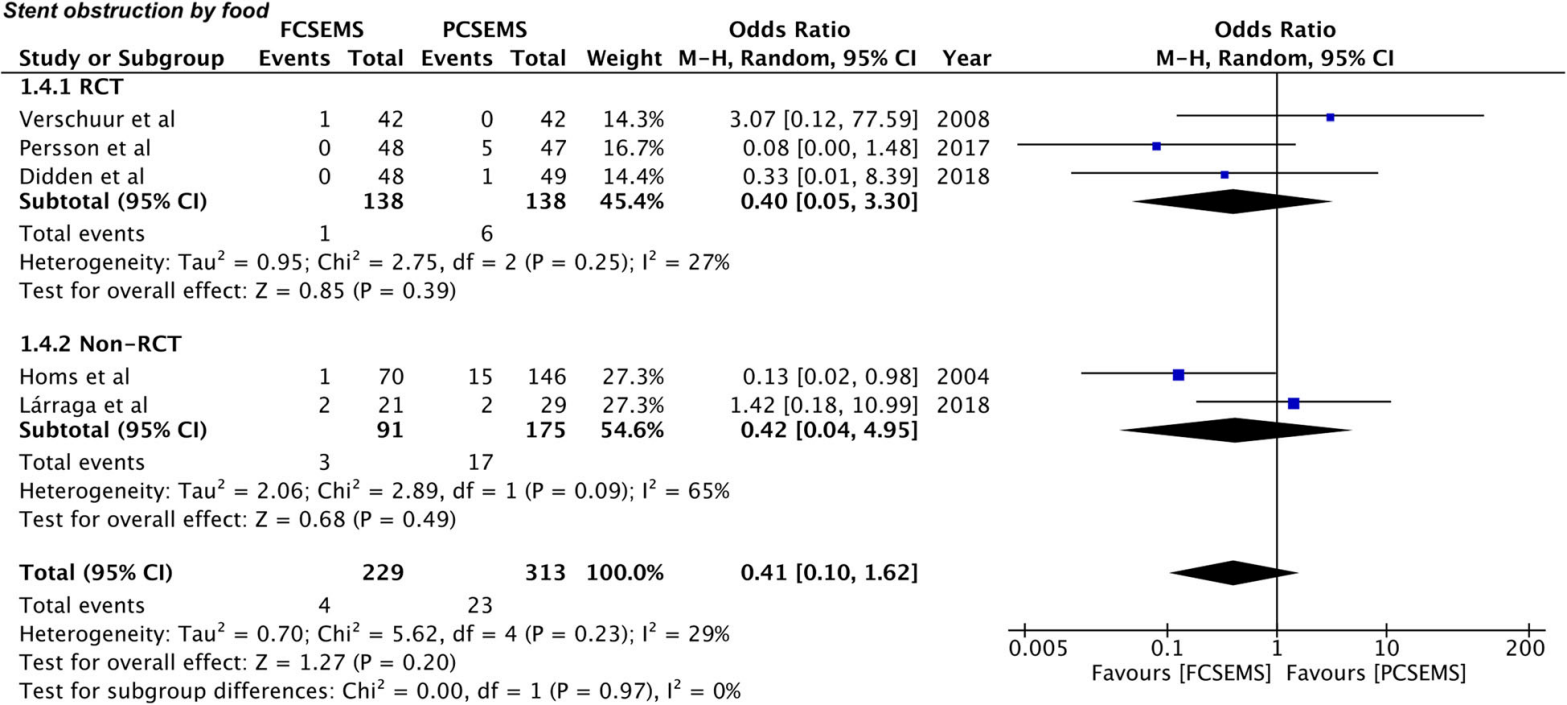
Forrest plot for stent obstruction by food

Incidence of stent obstruction by tissue growth or food impaction was also reported by all five included studies [26–30]. Incidence of stent obstruction due to tissue growth was 16.15% (37/229) in the FCSEMS group and 14.69% (46/313) in the PCSEMS group. Pooled analysis demonstrated no statistically significant difference between the two groups (OR 0.81, 95%CI 0.47–1.39, *P* = 0.44; I^2^ = 2%) (Fig. 4). Subgroup analysis for RCTs (OR 0.65, 95%CI 0.31–1.35, *P* = 0.25; I^2^ = 0%) and non-RCTs (OR 0.53, 95%CI 0.05–5.77, *P* = 0.61; I^2^ = 63%) also produced a similar result (Fig. 4). The incidence of stent obstruction by food was higher in PCSEMS (7.3%) as compared to FCSEMS (1.6%). However, the pooled effect remained statistically non-significant (OR 0.41, 95%CI 0.10–1.62, *P* = 0.20; I^2^ = 29%) (Fig. 5). Results for sub-group analysis of RCTs (OR 0.40, 95%CI 0.05–3.30, *P* = 0.39; I^2^ = 27%) and non-RCTs (OR 0.42, 95%CI 0.04–4.95, *P* = 0.15; I^2^ = 46%) were also non-significant (Fig. 5).

**Fig. 6:**
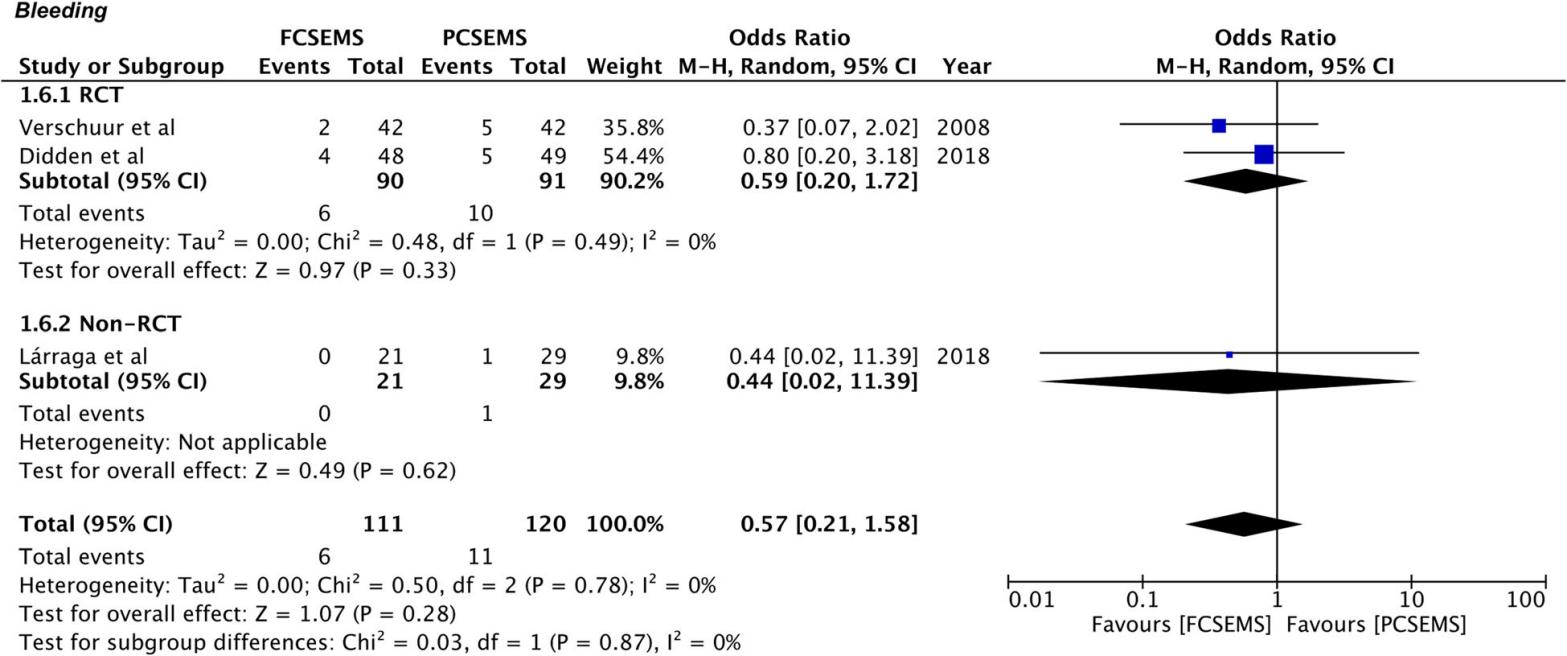
Forrest plot for bleeding

**Fig. 7:**
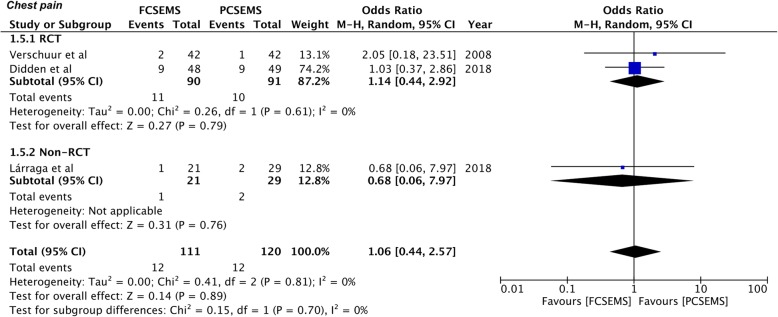
Forrest plot for chest pain

Since the definition of remaining complications varied across studies, only specific complications with sufficient available data were pooled for a meta-analysis. Data on post-operative bleeding and chest pain was available from three studies [27, 29, 30]. Our results demonstrate no statistically significant difference in the incidence of bleeding between the two groups (OR 0.57, 95%CI 0.21–1.58, *P* = 0.28; I^2^ = 0%) (Fig. 6). Similarly, there was no difference in the incidence of chest pain between FCSEMS and PCSEMS (OR 1.06, 95%CI 0.44–2.57, *P* = 0.89; I^2^ = 0%) (Fig. 7). No difference in results were noted in the sub-group analysis of RCTs and non-RCTs for both these complications (Figs. 6 & 7). On sensitivity analysis by sequential exclusion of individual studies, there was no change in the significance of results for any variable.

### Risk of bias assessment

The authors’ judgment of risk of bias assessment of RCTs is presented in Table 5. Adequate method of random sequence generation was followed by all three RCTs [27–29]. Allocation concealment [29] and blinding of participants [28] was reported by one trial each. Blinding of outcome assessment was not reported in any trial. Only one RCT was preregistered [29]. The Risk of bias assessment according to the RoBANS tool for non-RCTs is presented in Table 6.

**Table 5:** Risk of Bias summary for RCTs

Study	Random sequence generation	Allocation concealment	Blinding of participants and personnel	Blinding of outcome assessment	Incomplete outcome data	Selective reporting	Other Biases
Didden et al. [29]	Low risk	Low risk	High risk	High risk	Low risk	Low risk	Unclear risk
Persson et al. [28]	Low risk	Unclear risk	Low risk	High risk	High risk	Unclear risk	Unclear risk
Verschuur et al. [27]	Low risk	Unclear risk	High risk	High risk	High risk	Unclear risk	Low risk

**Table 6:** Risk of bias summary for Non-RCTs

Study	Selection of participants	Confounding variables	Intervention measurements	Blinding of outcome assessment	Incomplete outcome data	Selective outcome reporting
Lárraga et al. [30]	Low risk	High risk	Unclear risk	High risk	Low risk	Low risk
Homs et al. [26]	Low risk	Low risk	High risk	High risk	Low risk	Low risk

## Discussion

Owing to the limited comparative evidence between FCSEMS and PCSEMS, one of the primary objectives of this study was to compare the incidence of stent migration between the two devices. An important rationale of different design patterns of FCSEMS and PCSEMS was to reduce the incidence of migration with FCSEMS by leaving the proximal and distal flanges uncovered [34]. Stent migration is not only dependent on the stent design but also patient- related and surgical factors like the stent location, post-stenting chemotherapy or radiotherapy and use of clips or sutures [21, 30]. Migration rates are higher when stents are placed through the gastroesophageal junction as the lower end of the stent projects freely and unsupported in the fundus of the stomach [35]. Patients who undergo post-stenting chemotherapy or radiotherapy may also be prone to stent migration due to the reduction of the tumor size with adjuvant therapy. PCSEMS may be preferred by clinicians in such cases [3]. Baseline differences between the study groups for such confounding variables can introduce bias in the results of non-randomised studies. Another source of bias is the different types of SEMS used in the five studies of this review; with the greatest variation seen for FCSEMS. All four different types of FCSEMS used in the five studies have peculiar design elements to prevent stent migration. The European version of Z-stents are provided with one or two ring-shaped rows of barbs to prevent device migration [36]. The Niti-S stent flares to 26 mm at both ends and has an internal covering of polyurethane to allow the outer uncovered Niti wire to embed in the esophageal wall [37]. Wallflex FCSEMS also has a dog-bone shaped design with an internal covering. The outer wire framework implants itself in the tumor wall providing frictional resistance to dislocation [29, 38]. The fully covered SX-ELLA has a flip-flop type of anti-migration ring that is circumferentially attached to the proximal portion of the stent. The ring functions as a hook preventing stent migration [10, 30]. Considering the anti-migration design elements incorporated in all FCSEMS, it is not surprising that our analysis found no statistical significant difference in the migration rates of the two devices. Our results seem robust as there was no change in the effect size or direction after sensitivity analysis and sub-group analysis of RCTs and non-RCTs.

A meta-analysis for “improvement of dysphagia” could not be conducted due to difference in definitions and presentation of data. Several single-arm longitudinal studies have reported improvement in dysphagia scores with both FCSEMS and PCSEMS [7–9, 34]. Repici et al. [38] in a prospective multi-centre non-randomised study of 82 patients reported an improvement of dysphagia scores from a mean of 3 to a mean of 1 (*p* < 0.001) at 4 weeks following placement of Wallflex FCSEMS. Likewise, Saranovic et al. [39] have reported an improvement of dysphagia scores from 2.67 to 0.05 (on 0–4 scale) after 4 weeks, using the Ultraflex PCSEMS in 98 patients. Similar results were reported by all the four studies [27–30] reporting dysphagia outcomes in our review. Descriptive analysis of the four studies [27–30] suggests the difference in then basic design of FCSEMS and PCSEMS does not seem to have an impact on the improvement of dysphagia. Our results also indicate that; technical success, indicating successful placement of stent on the day of the planned procedure is not significantly different between the two SEMS. A very high technical success rate of 96.8% with FCSEMS and 96.4% with PCSEMS was pooled in our analysis.

Other than stent migration, stent obstruction due to tissue growth or food also results in recurrent dysphagia [39]. Tissue obstruction can be either due to either tumor growth or hyperplastic non-malignant overgrowth [37]. With the development of covered SEMS, the incidence of stent obstruction due to tumor ingrowth has reduced but this advantage is probably outweighed by the high rate of tissue overgrowth at the edge of the stents [10]. Tissue growth may also manifest through the uncovered proximal and distal edges of PCSEMS and therefore these stents may be more prone to obstruction as compared to FCSEMS. Seven et al. [21] in a retrospective study of 252 patients with benign and malignant esophageal lesions have reported ﻿a higher incidence of tissue ingrowth or outgrowth with PCSEMS as compared to FCSEMS (53.4 vs. 29.1%, *p* = 0.004). The groups were however not matched with a greater number of malignant lesions treated with PCSEMS (*p* < 0.001). The results of our meta-analysis indicate that there is no difference between the two devices for rates of stent obstruction with tissue growth. The results were similar for both RCTs and non-RCTs. It has been suggested that while using PCSEMS, the selection of stent size should be based on the length of the covering rather than the complete length of the stent [27]. Overlaying the entire tumor length with the covered portion of PCSEMS may prevent malignant tissue ingrowth thereby reducing obstruction. The absence of any difference between FCSEMS and PCSEMS in our review may have been influenced by the stent size used in the individual studies.

The obstruction of the stent due to food has been attributed to a lack of peristalsis and fixed diameter of the stent lumen. Blockage usually occurs due to discrepancy in the size of the bolus and lumen of the stent or adherence of food to defects in the stent covering or in the uncovered portion of PCSEMS [26]. This may be one of the reasons for higher incidence of food obstruction seen with PCSEMS in our pooled analysis. However, the difference was not statistically significant. In addition to stent related factors, patient compliance is important to prevent food obstruction. Clear and specific instruction to patients on having liquids between meals to flush the food and through chewing of food helps reduce the rate of food impaction [4]. For other complications, data only for chest pain and bleeding was pooled in our analysis. Retro-sternal pain after placement of SEMS has been attributed to the high axial force resulting in pressure on the malignant lesion [29]. Our analysis indicated that there is no difference between the two devices in terms of chest pain and bleeding. It is important to note that evidence is limited, as only three studies [27, 29, 30] were pooled for these variables.

Some limitations of our study need to be elaborated. Firstly, the overall quality of the included studies was not high. The risk of bias in individual studies may have compromised the level of evidence of our review. Secondly, only three RCTs [27–29] were available for inclusion in the review. Non-randomised studies are prone to bias and may have influenced results. Thirdly, a variety of different stents with different design characteristics were used in the five trials. The influence of specific stent design on the overall outcome cannot be disregarded. Lastly, a meta-analysis on improvement of dysphagia and total overall complications was not possible due to the heterogeneity of the included studies.

Nevertheless, our study is the first meta-analysis comparing FCSEMS and PCSEMS for malignant esophageal lesions. The stability of results on sensitivity analysis and sub-group analysis of RCTs and non-RCTs lends credibility to the inferences of our study.

## Conclusions

To conclude, our results indicate that there is no difference in FCSEMS and PCSEMS in terms of successful stent placement, stent migration and stent obstruction when used for palliative treatment of inoperable esophageal malignancy. The quality of evidence is however weak. In line with our results, it may be suggested that surgeons managing esophageal cancer may use any of the two stents without any difference in overall outcomes. However, in our opinion, individual patient characteristics and surgeon preference should continue to guide stent selection in patients with inoperable esophageal cancer.

## Data Availability

The datasets used and/or analyzed during the current study are available from the corresponding author on reasonable request.

## Abbreviations

CENTRAL: Cochrane Central Register of Controlled Trials
CI: Confidence Interval
FCSEMS: Fully covered self-expanding metallic stents
OR: Odds ratio
PCSEMS: Partially covered self-expanding metallic stents
RCT: Randomised controlled trial
RoBANS: Risk of bias assessment tool for non-randomized studies
SEMS: Self-expanding metal stents
